# Classification of rice plant diseases using efficient DenseNet121

**DOI:** 10.1038/s41598-026-38078-6

**Published:** 2026-02-20

**Authors:** Amr Ismail, Walid Hamdy, Ali H. Ibrahim, Wael A. Awad

**Affiliations:** 1 Faculty of Science, Port Said University, Port Said, Egypt; 2 Faculty of Computers and Artificial Intelligence, Misr University for Science & Technology, Giza, Egypt; 3 Faculty of Computers and Artificial Intelligence, Dameitta University, New Damietta, Egypt

**Keywords:** DenseNet121, Rice plant disease, CNN, Classification, Machine learning, Plant sciences, Mathematics and computing

## Abstract

Agriculture and global food security are critically dependent on accurate and timely identification of plant diseases and pests. Traditional approaches to disease identification rely heavily on visual inspection and expert knowledge, which frequently lack the accuracy, speed, and scalability needed to address growing agricultural challenges. Early and precise disease detection enables proactive interventions that can prevent widespread crop damage and reduce excessive pesticide use, thereby supporting sustainable agricultural practices. Artificial intelligence, particularly deep learning methods, has emerged as a transformative solution for automated plant disease diagnosis. Convolutional neural networks (CNNs) have demonstrated remarkable capabilities in image classification tasks, evolving from individual architectures to sophisticated ensembles and transferring learning models. However, existing CNN-based research on rice disease identification has typically focused on a limited number of disease classes, restricting their practical applicability in real-world agricultural settings. This study addresses these limitations by implementing DenseNet121, an advanced CNN architecture known for its efficient feature reuse and gradient flow, for comprehensive rice disease classification. We utilized a dataset comprising seven of the most common rice diseases, significantly expanding the scope beyond previous studies. The model employs transfer learning with pre-trained ImageNet weights and is optimized using the Adam optimizer with carefully tuned hyperparameters. The experimental evaluation on an independent test set demonstrates that our proposed model achieves an overall accuracy of 97.9%, with individual disease classification accuracy ranging from 94% to 99.67%. The model exhibits balanced performance across multiple metrics, including precision (96.2%), recall (97.97%), and F1-score (97%), confirming its robustness and generalizability. These results establish DenseNet121 as a highly effective framework for automated rice disease diagnosis, offering a practical tool for enhancing agricultural productivity and food security.

## Introduction

 Plant diseases are becoming more common, which is a major danger to food security, economic stability, and environmental sustainability in the agricultural sector worldwide. The need for agricultural production is expected to rise significantly as the world population continues to grow, with projections indicating that it will approach 10 billion people by 2050. Thus, there is a greater need than ever for efficient disease management techniques. Conventional techniques for identifying and managing plant diseases are labor-intensive and prone to human error since they frequently rely on visual inspections and specialist expertise. Therefore, novel approaches that can improve the precision and effectiveness of plant disease detection are desperately needed^1^.

More than 7 billion people can today be fed on the food that human society produces. Food security is still threatened by a number of reasons, though, including plant diseases, climate change, and the loss of pollinators^2^.

In addition to posing a threat to global food security, plant diseases have the potential to prove disastrous for smallholder farmers whose livelihoods depend on producing healthy crops. In emerging nations, almost 80% of agricultural output is produced by smallholder farmers^3^. Moreover, they comprise half of the world’s hungry, rendering them especially vulnerable to disruptions in the food supply caused by pathogens^4^. It is imperative to halt the proliferation of the disease at its initial stage, as numerous reports indicate crop losses exceeding 50% due to pests and diseases^5^. The identification of plant diseases has become more accessible due to the proliferation of smartphones and deep learning advancements. However, in order to build reliable image classifiers for plant disease diagnosis, a sizable, validated dataset of photos of both sick and healthy plants is required. Up until recently, there was no such dataset, and even smaller datasets were not publicly accessible^6^. The application of computer vision technology to the assessment of rice (*Oryza sativa*) grades and quality management has grown in popularity. In order to measure the quality of rice, a variety of image-processing approaches have been presented. These techniques include size and shape^3^, color analysis, root and shoot length estimation, broken ratio, whiteness, and fissure identification. While these image-processing methods produce improved outcomes, they all share one drawback: because they necessitate sophisticated feature extraction and picture pre-processing methods, the algorithms reduce the effectiveness of real-time detection^7^.

An increasing number of people are interested in using Artificial Intelligence (AI), specifically machine learning and deep learning methods, to improve agricultural pest and disease identification. A branch of AI known as “machine learning” involves teaching machines to identify patterns and make predictions using massive datasets. One effective area of machine learning is deep learning. Multiple-layer neural network training is involved^8^. It has made digital image processing much more sophisticated. Due to this advancement, it is now more often used to identify pests and plant diseases, which has made it a popular area of study^9^. In this context, a neural network recognizes and outputs the particular crop disease from an image of a diseased plant that it has received as input. A major issue, though, is developing a deep network that efficiently maps inputs to outputs while taking its topology into account. Deep neural networks are trained, and the network parameters are changed over time to better this mapping. In order to increase performance, a number of theoretical and practical developments have been made in this intricate computational process^10^.

Automated plant disease diagnosis now has more options because of recent developments in machine learning and computer vision. Convolutional neural networks (CNNs) are one of the sophisticated machine learning architectures that have been developed for picture classification applications, such as the identification of plant diseases^11^. Densely Connected Convolutional Networks (DenseNet) (more precisely, DenseNet121) is one of the most promising CNN designs; it has shown remarkable performance across a range of image classification benchmarks^12^. Direct connections between layers are made possible by DenseNet distinctive architecture, which enhances gradient flow and feature reuse. By reducing the number of parameters, this architecture improves the model computational efficiency while also improving its capacity to learn complicated representations. Plant disease detection is a relatively new field in which the DenseNet121 program is being used, but it has great promise. Through the utilization of this architecture advantages, scientists may create models that reliably recognize and categorize an extensive array of plant diseases based on leaf photos^13^. By drastically cutting down on the time and resources needed for disease identification, these automated devices could help farmers and other agricultural experts respond to problems in a timely manner. Moreover, the incorporation of machine learning into agricultural methods is consistent with the precision agriculture movement, which aims to maximize inputs and outputs by means of data-driven decision-making.

The importance of plant disease detection goes beyond the health of a single crop to include larger implications for food security and agricultural sustainability^14^. Plant diseases can have a disastrous economic impact by lowering crop yields, raising production costs, and eventually creating a food shortage. For example, according to estimates from the Food and Agriculture Organization (FAO), plant diseases cause 20–40% of crop losses worldwide each year. This startling figure highlights the need for efficient disease control plans that might lessen these losses and guarantee the long-term viability of agricultural systems^15^.

Several studies have investigated the use of deep learning methods, such as DenseNet, in the field of plant disease identification in recent years. This research has shown that classifying diseases in rice crops, like bacterial leaf blight, bacterial leaf streak, etc. may be accomplished with CNNs. The findings are encouraging, showing that deep learning models can identify diseases with high accuracy rates, frequently outperforming conventional techniques. Nevertheless, implementing these models in actual agricultural environments has a distinct set of difficulties^16^. To achieve successful adoption, issues including the necessity for model interpretability, the availability of high-quality labeled datasets, and the integration of these technologies into current agricultural practices must be addressed.

Moreover, creating a model that is broadly applicable is made more difficult by the variety of plant species and the unpredictability of disease symptoms. Distinct disease presentations are seen in different crops, and these symptoms can also be influenced by environmental factors. As such, it is critical to customize models to particular crops and diseases while accounting for the particulars of each situation. To develop reliable and flexible models that can meet the many demands of the agricultural industry, agronomists, plant pathologists, and data scientists must continue their research and work together^17^. The current study uses the DenseNet121 model to categorize different plant diseases based on leaf photos in an effort to add to the expanding body of knowledge on plant disease identification. The objective of this research is to offer insights into the effectiveness of deep learning techniques in agricultural applications by assessing the model performance and contrasting it with alternative machine learning methodologies. In addition, the research will tackle the difficulties related to model training, dataset preparation, and assessment metrics, adding to the current integration of AI application in agriculture^18^.

In summary, there is potential to improve plant disease identification and control at the nexus of machine learning and agriculture. The DenseNet121 model provides a workable answer to the urgent problems the agriculture industry is facing by leveraging its sophisticated architecture and demonstrated capabilities. To address the global concerns of environmental sustainability and food security, developing and applying cutting-edge agricultural technologies will be crucial^19^. The goal of this research is to provide insight into how deep learning may improve plant disease management strategies, thereby enhancing the sustainability and resilience of global food systems.

## Related work

Plant disease classification has garnered significant attention in recent years as agriculture faces the dual challenges of increasing global food demand and the impacts of climate change. This section provides a thorough overview of relevant research papers that have been published so far on sustainable agriculture, with an emphasis on new developments in crop pest and disease classification and detection.

Deep learning, especially through the use of convolutional neural networks (CNNs), has transformed how scientists tackle plant disease identification and classification, facilitating the development of highly accurate and efficient automated detection systems^20^. Among the various CNN architectures, DenseNet (Densely Connected Convolutional Networks) has emerged as a powerful tool due to its intricate structure that connects each layer to every other layer in a feed-forward manner^21^. The foundational work by Mohanty et al., who utilized CNNs for plant disease identification, set a precedent for further exploration in the field^20^. They developed a model that achieved remarkable accuracy in classifying images of leaves affected by various diseases. This work underscored the potential of neural networks to analyze high-dimensional data typical of leaf imagery, paving the way for subsequent studies that built upon these methods. DenseNet, introduced by Huang et al., offers significant advantages over traditional CNN architectures^21^. The key innovation of DenseNet is its dense connectivity, which allows for better gradient flow during training and reduces the number of parameters, ultimately lessening the risk of overfitting^21^. Studies such as those have highlighted the efficacy of DenseNet in medical image classification^22^, indicating its adaptability to various imaging tasks, including the classification of diseased plant leaves. This adaptability can be attributed to DenseNet efficient use of features through its dense connectivity, which encourages feature reuse across layers.

Numerous studies have specifically focused on applying DenseNet models for plant disease classification. For instance, Sultana et al.^23^. employed a modified DenseNet architecture, fine-tuning it on a dataset of images of diseased plants. Their approach demonstrated that DenseNet not only outperformed traditional models in terms of accuracy but also showed improved computational efficiency due to its fewer parameters. Additionally, the authors emphasized the importance of data augmentation techniques in enhancing the robustness of their model, which is a common practice in the domain of image classification^24^. Another noteworthy study explored the implementation of DenseNet in a comprehensive framework for plant disease diagnosis^25^. They integrated the DenseNet architecture with transfer learning, leveraging pre-trained models to enhance performance on smaller datasets typical in agricultural research. Their findings confirmed the efficacy of transfer learning, with the DenseNet model achieving superior classification performance compared to other architectures, thus reinforcing the growing consensus regarding the benefits of leveraging pre-trained models in specialized domains such as plant pathology^26^.

Plant disease classification is essential for ensuring food security and global agriculture. There is an urgent need to develop strong approaches for the correct identification and categorization of plant diseases due to the growing threat posed by invasive pathogens and the consequences of climate change. This literature review covers the categorization of plant diseases and the state of the art in this field, including established techniques, developments in computer vision and machine learning, and difficulties faced by researchers.

Traditional methods for plant disease diagnosis have relied heavily on visual inspection and expert knowledge. According to Savary et al., these conventional techniques are time-consuming and often limited by diagnostic expertise^27^. The reliance on human interpretation can lead to variability in disease detection, underscoring the necessity for more objective and automated systems. Early attempts at digital image analysis aimed to enhance traditional diagnostic processes. For instance, Fradgley et al., demonstrates the potential of image processing techniques in identifying symptoms of diseases such as powdery mildew on cereal crops^28^. However, limitations in the reliability of image analysis under varying environmental conditions have been noted, necessitating further research and development.

While the application of machine learning has yielded promising results, several challenges persist in the classification of plant diseases. One critical issue is the need for large and diverse datasets to train models effectively. Current datasets may not adequately represent the variability in plant species, diseases, and environmental conditions, leading to overfitting and decreased model generalizability^29^. Moreover, the annotation process of images can be labor-intensive and costly, further complicating the creation of robust datasets^30^. Crowdsourcing and citizen science initiatives have been proposed as potential solutions to enrich datasets; however, the quality and reliability of such data are often questioned^31^.

Another significant hurdle in plant disease classification is the presence of simultaneous infections, where multiple pathogens affect the same plant. This condition complicates visual symptom assessment and can lead to misclassification^32^. To address this, multi-label classification approaches are gaining traction, allowing models to predict multiple disease classes simultaneously. Wang et al. explored these methods, demonstrating that incorporating multi-label strategies can enhance diagnostic accuracy in scenarios with co-infection^33^.

Furthermore, the deployment of machine learning models in real-world agricultural settings introduces additional complexities, including the need for real-time processing, minimal computational resources, and mobile applicability^34^. Researchers are increasingly focusing on simplifying model architectures, optimizing for lower computational power while maintaining accuracy. Transfer learning has emerged as a viable strategy, enabling models trained on large datasets to be fine-tuned for specific applications with limited data^35^. In^36^ they focus on detecting diseases in rice leaves using deep learning techniques. To classify diseases in rice leaves using DenseNet architectures (DenseNet121, DenseNet169, and DenseNet201) and evaluate both accuracy and training speed. They achieved accuracy DenseNet121: 94%, DenseNet169: 89% and DenseNet201: 92%. DenseNet121 demonstrated the best combination of accuracy and efficiency.

Kunduracioglu studied the performance of several deep CNN architectures in classifying apple leaf diseases^37^. The study compared well-known models, such as ResNet50, InceptionV4, Xception, DenseNet121, EfficientNetV2_m, and VGG13, using conventional performance metrics like accuracy, precision, recall, and F1-score. Experimental results showed that all models performed well in classification, with EfficientNetV2_m outperforming the other designs with its highest accuracy and F1-score of 100%. These findings validate the ability of deep learning models to detect plant diseases accurately, as well as their potential to enable early diagnosis and decision-making in smart agriculture systems. For example, a comprehensive study was reported that used advanced deep-learning techniques to diagnose grape leaf diseases and even identify grape leaf varietals^38^. They assessed 14 CNN and 17 vision-transformer models and found very high performance: four models reached 100% accuracy in both disease classification and leaf-type recognition tests, with Swinv2-Base standing out for its remarkable results. That study shows that contemporary deep-learning architectures, rather than traditional CNNs, can be highly effective for plant disease diagnosis and varietal identification, pointing to a potential future for automated agricultural diagnostics and crop monitoring systems.

Kunduracioglu investigated, the efficacy of various residual network (ResNet) designs for disease detection in tomato leaves^39^. The scientists fine-tune multiple ResNet-based deep learning models on a labeled dataset of tomato-leaf images before rigorously evaluating their performance in detecting damaged versus healthy leaves. Their findings show that these ResNet-based models perform well in classification, showing that deep convolutional networks are a viable and practical way to automate plant disease detection in tomato crops. This study adds to the expanding body of research demonstrating that modern deep-learning approaches can provide speedy, accurate, and scalable disease diagnosis in agriculture. Finally, Kunduracıoglu and Paçal researchers evaluated CNN architectures from the EfficientNet-v1 and v2 families to detect diseases in sugarcane leaves^40^. The study used a publicly available dataset of 6,748 images from 11 disease classes. They also compared the performance of the EfficientNet variations to other prominent CNN models. The results demonstrated that neither more model complexity nor deeper architecture necessarily correlates with higher classification accuracy. Among 13 tested models, EfficientNet-b6 and InceptionV4 attained the best accuracies (~ 93.39 and ~ 93.10, respectively). These findings highlight the potential of deep-learning techniques for rapid and reliable disease diagnosis in sugarcane, adding to larger initiatives in smart agriculture for automated plant disease detection and crop management.

## Methodology

In this section, we describe the approach used in this study to construct and run experiments using DenseNet121 to classify plant disease datasets. The overall goal of our research is to improve the accuracy and efficiency of plant disease detection, which is critical for agricultural productivity and sustainability. Our methodology involves data collection, preprocessing, model creation, training procedures, evaluation metrics, and experimental settings. As demonstrated previously^41^, the classification, detection, and segmentation tasks have been addressed. It underlines the dominance of CNN-based models, the increasing usage of vision transformers, and the need for more diversified datasets and real-world validations.

### Dataset description

**Fig. 1:**
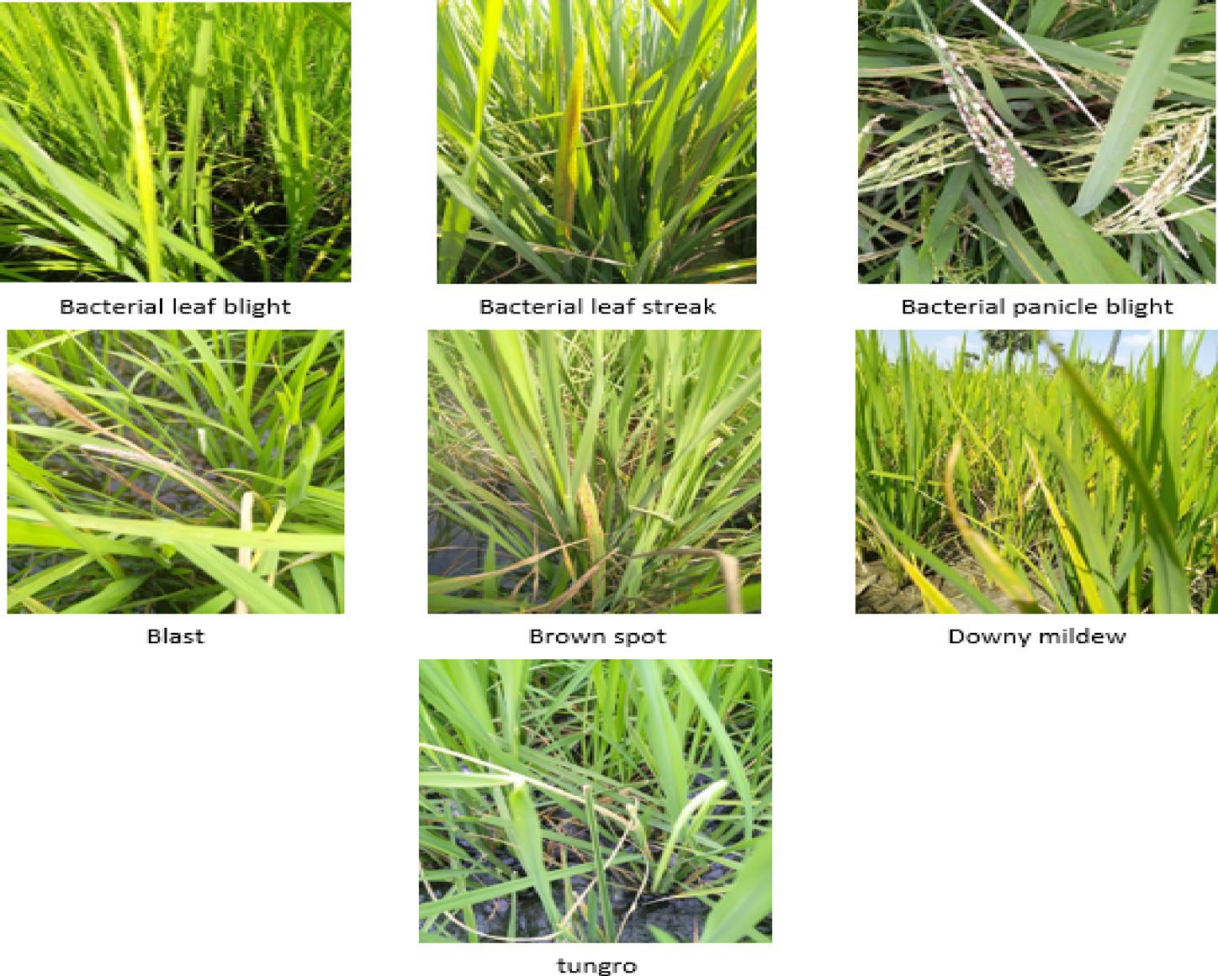
Sample of paddy-rice dataset.

In our experiments, we used a Paddy-Rice Dataset^42^, an open-source dataset for crop diseases that addresses the critical need for early identification of plant pests and diseases, which can significantly impact agricultural production and food security. The dataset consists of 8030 images. These images are associated with seven distinct classes of different rice diseases, as shown in Table 1. The categories of several rice diseases include bacterial leaf streak, brown spot, tungro, bacterial leaf blight, blast, downy mildew, and bacterial panicle blight. Figure 1 illustrates a sample of the dataset utilized in our investigation.

**Table 1:** Number of paddy-rice dataset samples.

Diseases classes	Number of images
Bacterial leaf blight	648
Downy mildew	868
Tungro	1951
Blast	2351
Brown spot	1257
Bacterial panicle blight	450
Bacterial leaf streak	505
**Total of images**	8030

### Data augmentation

To improve the model’s robustness and avoid overfitting, various data augmentation methods were employed during the training process. The techniques incorporated random rotation, zooming, horizontal and vertical flips, and adjustments to image brightness. The augmentation was applied in real-time during the training process to create a diverse set of training images. After augmentation, as seen in Table 2, we obtained 11,467 images.

**Table 2:** Number of paddy-rice dataset samples after augmentation.

Diseases classes	Number of images
Bacterial leaf blight	1238
Downy mildew	1360
Tungro	2257
Blast	2648
Brown spot	1574
Bacterial panicle blight	1120
Bacterial leaf streak	1270
**Total of images**	11,467

This study used a collection of 11,467 Paddy-Rice images from 7 categories, for different rice plants diseases. The dataset provides strong support for model training and performance evaluation. To ensure optimal training, stability, and generalization, the dataset was divided into two subsets: 80% training and 20% testing. A randomized sampling technique was used to reduce manual bias and assure fairness and randomness in the experimental outcomes. During partitioning, special care was taken to ensure class balance by carefully selecting and distributing samples from each category among the subsets. This strategy ensured a representative and homogeneous class distribution, allowing for fair model evaluation and quick training. The rice disease experimental dataset is summarized in Table 3.

**Table 3:** Data on different categories of paddy-rice dataset.

Diseases classes	Training set	Test set	Total
Bacterial leaf blight	866	372	1238
Downy mildew	952	408	1360
Tungro	1580	677	2257
Blast	1853	795	2648
Brown spot	1102	472	1574
Bacterial panicle blight	784	336	1120
Bacterial leaf streak	889	381	1270
**Total of images**	8026	3441	11,467

### Proposed model

The diagnosis of plant diseases is crucial for food security and agriculture. Proactive actions can be implemented to stop the spread of infestations and reduce the need for heavy pesticide use by enabling the early detection and classification of diseases. The suggested model uses DenseNet121 for classification in an effort to reliably identify plant diseases. The model aims to efficiently and accurately categorize a variety of crop-related photos utilizing images from the Crop Disease Detection Dataset.

**Fig. 2:**
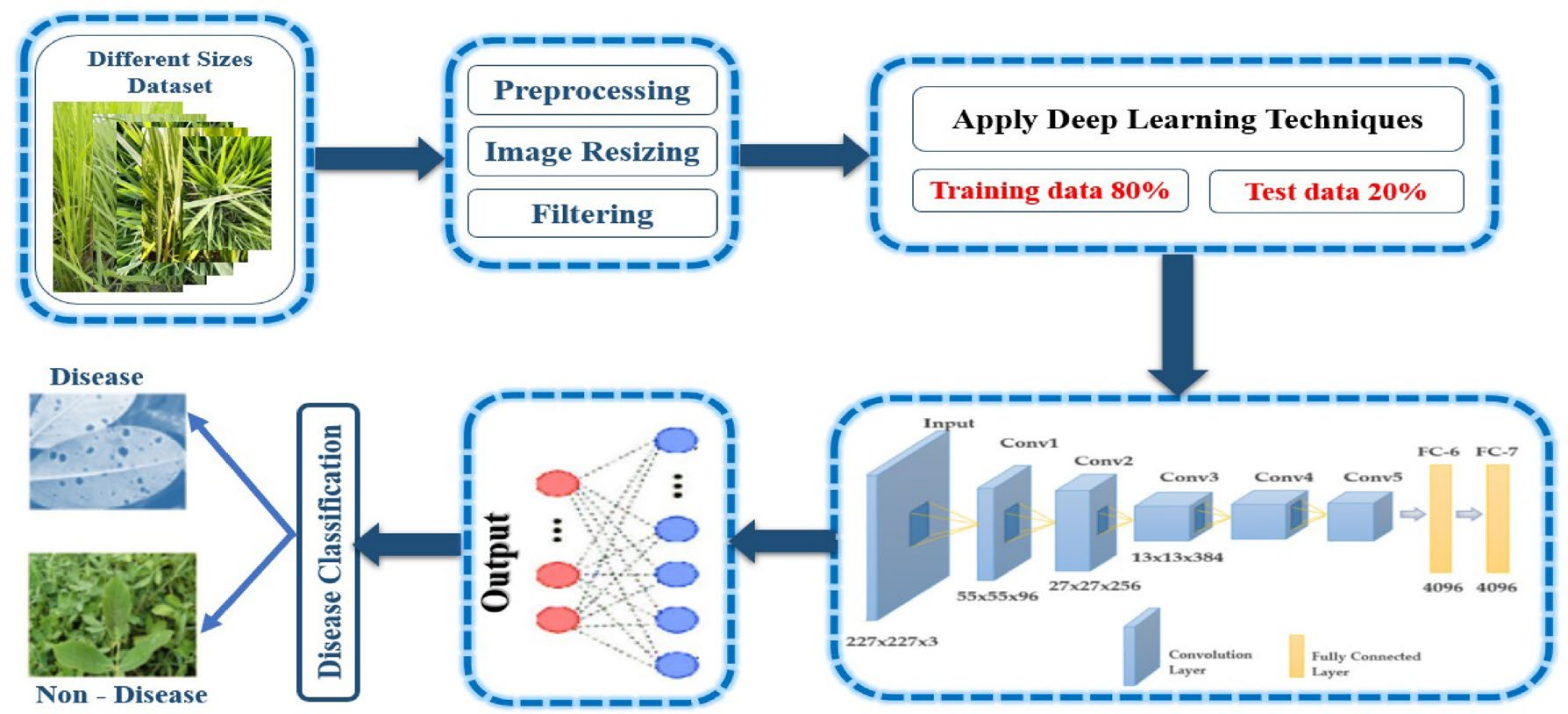
General architecture of our deep learning models.

Our proposed overall architecture of deep learning models is shown in Fig. 2, which consists of input datasets, preprocessing, deep learning models, transfer learning, classification, and evaluation metrics phases. Initially, the proposed deep learning models can be used to detect and reveal rice leaf diseases. The classification of plant diseases is the second phase. It takes a lot of time to train a neural network from the beginning. It requires the application of a sound hyperparameter selection method. Alternatively, it is easier and produces superior categorization performance metrics to transfer the weights from a traditional pre-trained network.

**Fig. 3:**
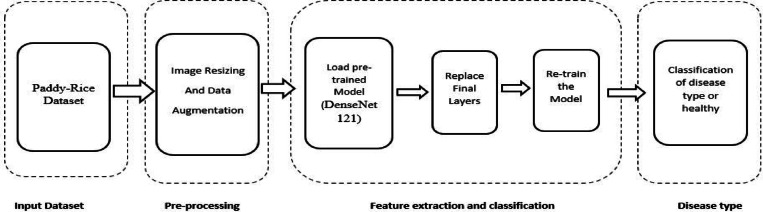
Transfer learning-based classification.

Figure 3 shows the schematic diagram for the transfer learning-based rice plant disease categorization based on the disease images. The images of diseases are adjusted in size to fit the standard input dimensions required by a pre-trained neural network. To enhance the network performance, since deep neural networks benefit from larger datasets, rotation-based data augmentation is applied during the pre-processing phase. The network weights and first layers of the chosen model are moved. From the photos of the diseases, the relevant network extracts the discriminative properties. Changing the final layers makes classification possible.

### DenseNet121 architecture

The DenseNet121 architecture, a convolutional neural network (CNN) known for its efficiency and effectiveness in image classification tasks, was selected for this study. DenseNet121 consists of 121 layers and employs a unique connectivity pattern, where each layer receives inputs from all preceding layers. This architecture promotes feature reuse, reduces the number of parameters, and mitigates the vanishing gradient problem.

The model was set up with weights pre-trained on the ImageNet dataset, enabling it to utilize features learned from a vast and varied collection of images. The final classification layer was modified to match the number of classes in the plant disease datasets. Specifically, for the Paddy-Rice dataset, the output layer was adjusted to classify between 12 disease categories.

Finally, the architecture of DenseNet121 consists of:

- **Total Layers**: 121 layers organized in dense blocks.
- **Dense Blocks**: 4 dense blocks with^6,11,15,23^ layers respectively.
- **Transition Layers**: Between dense blocks for dimensionality reduction.
- **Growth Rate**: 32 (number of feature maps added per layer).
- **Total Parameters**: ~8 million (vs. ~138 million for VGG16).

**Key Architectural Advantages**:

1. **Dense Connectivity**: Each layer receives inputs from all preceding layers, promoting feature reuse.
2. **Gradient Flow**: Direct connections alleviate vanishing gradient problem.
3. **Parameter Efficiency**: Fewer parameters than comparable architectures (ResNet, VGG).
4. **Feature Propagation**: Encourages feature map reuse throughout the network.

#### Transfer learning configuration

**Pre-trained Weights**:

- Source: ImageNet dataset (1.2 M images, 1,000 classes).
- Pre-trained layers: All convolutional layers in dense blocks.
- Weight initialization: Xavier/Glorot uniform for new layers.

**Model Modification for Rice Disease Classification**:

1. **Frozen Layers (Initial Training Phase)**:

- All convolutional layers in dense blocks 1–3 frozen.
- Only dense block 4 and classification layers trainable.
- Purpose: Preserve low-level feature representations learned from ImageNet.

2. **Fine-tuning Phase**:

- Gradually unfroze dense blocks 3 and 4.
- Continued training with lower learning rate (0.0001).
- Purpose: Adapt mid-level features to rice disease characteristics.

3. **Classification Head Modification**:

- Removed original 1,000-class classification layer.
- Added custom classification head:
  - Global Average Pooling (GAP) layer.
  - Dropout layer (rate: 0.5) for regularization.
  - Dense layer with 256 units + ReLU activation.
  - Dropout layer (rate: 0.3).
  - Output Dense layer with 12 units + Softmax activation.

### Training procedure

The training procedure involved several key steps, including the definition of loss functions, optimization algorithms, and hyperparameter tuning.

4. **Loss Function**: The categorical cross-entropy loss function was utilized, as it is particularly effective for addressing multi-class classification challenges. This loss function quantifies the discrepancy between the model predicted probabilities and the actual class labels, directing the model to refine its predictions accordingly.
5. **Optimization Algorithm**: The Adam optimizer was selected for training the DenseNet121 model. Adam is an optimization algorithm that adaptively adjusts the learning rate for each parameter by integrating the strengths of two other enhancements to stochastic gradient descent. It has been shown to converge faster and achieve better performance in various deep learning tasks.
6. **Learning Rate Scheduling**: A learning rate scheduler was introduced to dynamically modify the learning rate throughout training. The starting learning rate was established at 0.001, and a ReduceLROnPlateau scheduler was applied to lower the learning rate by a factor of 0.1 whenever the validation loss failed to improve over three consecutive epochs.
7. **Batch Size and Epochs**: The batch size was set to 32, allowing for efficient utilization of GPU memory while maintaining stable gradient estimates. The model was trained for a maximum of 50 epochs, with early stopping implemented to halt training if the validation loss did not improve for five consecutive epochs.
8. **Training Process**: The training process was carried out in a high-performance computing environment. Each training iteration involved feeding a batch of images through the model, computing the loss, and updating the model weights using backpropagation. The training and validation losses were monitored throughout the process to ensure that the model was learning effectively.

## Experimental results and discussion

To assess the performance of the DenseNet121 model on the plant disease datasets, several evaluation metrics were utilized:

**The sensitivity**, Recall, also known as true positive, refers to the accuracy of positive instances and the number of correctly labeled examples of positive sets. It can be calculated using Eq. (1), where TP (true positive) refers to the count of positive instances that are correctly identified by the classifier, while FN (false negative) represents the number of positive instances that are mistakenly labeled as negative

1 $$\:\mathrm{S}\mathrm{e}\mathrm{n}\mathrm{s}\mathrm{i}\mathrm{t}\mathrm{i}\mathrm{v}\mathrm{i}\mathrm{t}\mathrm{y}\left(\mathrm{R}\mathrm{e}\mathrm{c}\mathrm{a}\mathrm{l}\mathrm{l}\right)=\frac{TP}{TP+FN}$$

**Specificity** can be defined as the restrictive probability of actual negatives token an optional class, which generally translates to the likelihood that the negative marking is true. This can be expressed using Eq. (2), where FP denotes the number of false positives or cases that are incorrectly assigned as positive and TN represents the number of cases or real negatives that are negative and named as such.

2 $$\:\mathrm{S}\mathrm{p}\mathrm{e}\mathrm{c}\mathrm{i}\mathrm{f}\mathrm{i}\mathrm{c}\mathrm{i}\mathrm{t}\mathrm{y}=\frac{TN}{TN+FP}$$

Generally speaking, sensitivity and accuracy are either good or negative indicators of how well an algorithm performs for a certain class.

The most common criterion used to evaluate classification efficiency is precision. Every 20 iterations during the assessment period, the accuracy was assessed. Equation (3) represents this metric, which counts the percentage of samples that are correctly classified.

3 $$\:\mathrm{A}\mathrm{c}\mathrm{c}\mathrm{u}\mathrm{r}\mathrm{a}\mathrm{c}\mathrm{y}=\frac{TP+TN}{\mathrm{T}\mathrm{P}+\mathrm{T}\mathrm{N}+\mathrm{F}\mathrm{P}+\mathrm{F}\mathrm{N}}$$

**Precision** is obtained using Eq. (4), which divides the total number of true positives by the sum of the true positives and the false positives. This metric assesses how accurate the algorithm is at foreseeing outcomes. The precision of the model is determined by how “exact” it is in terms of the proportion of expected positives that actually occur.

4 $$\:\mathrm{P}\mathrm{r}\mathrm{e}\mathrm{c}\mathrm{i}\mathrm{s}\mathrm{i}\mathrm{o}\mathrm{n}=\frac{\mathrm{T}\mathrm{P}}{\mathrm{T}\mathrm{P}+\mathrm{F}\mathrm{P}}$$

### Results

A DenseNet121 performance can be enhanced by carefully choosing hyperparameters such as batch size, maximum epochs, and step size. The pre-trained models are trained with a batch size of 32. When applying a pre-trained network to a new task through transfer learning, using a small step size (learning rate) such as 0.00001 and training for 50 epochs can lead to enhanced network performance. Moreover, the methodology outlined in this section details the comprehensive approach taken to design and execute experiments utilizing DenseNet121 for plant disease classification. Through careful dataset preparation, model architecture selection, training procedures, evaluation metrics, and experimental setup, we aimed to achieve robust and reliable results that contribute to the field of agricultural technology and plant pathology. The subsequent sections will present the results obtained from these experiments, along with a discussion of their implications and potential future directions for research. The input layer of DenseNet121 processes every individual image. The split between training and testing data was randomly determined, with a typical ratio ranging from 80% for training to 20% for testing. Table 4 shows the outperformed pre-trained DenseNet121 for each type of rice disease.

**Table 4:** Illustration and evaluation of each type of rice disease.

Disease Types	Sensitivity	Specificity	Precision	F-score	Accuracy
Bacterial leaf blight	99.97%	96%	97%	97%	99%
Bacterial leaf streak	99%	96%	96%	94%	99%
Bacterial panicle blight	99.97%	100.00%	99.67%	99.52%	99.90%
Blast	96%	97%	96%	97%	99%
Brown spot	97%	96%	94%	96%	99%
Downy mildew	99%	97%	96%	99%	99%
Tungro	99%	96%	99%	99%	96%

The final results of our proposed model are shown in Table 3. The proposed model obtained the superior average accuracy, DenseNet-121 had an accuracy of 97.9% as shown in Table 5.

**Table 5:** Illustrate the final result of rice disease.

Disease Types	Sensitivity	Specificity	Precision	F-score	Accuracy
Our proposed model	97.97%	96.6%	96.2%	97%	97.9%

**Fig. 4:**
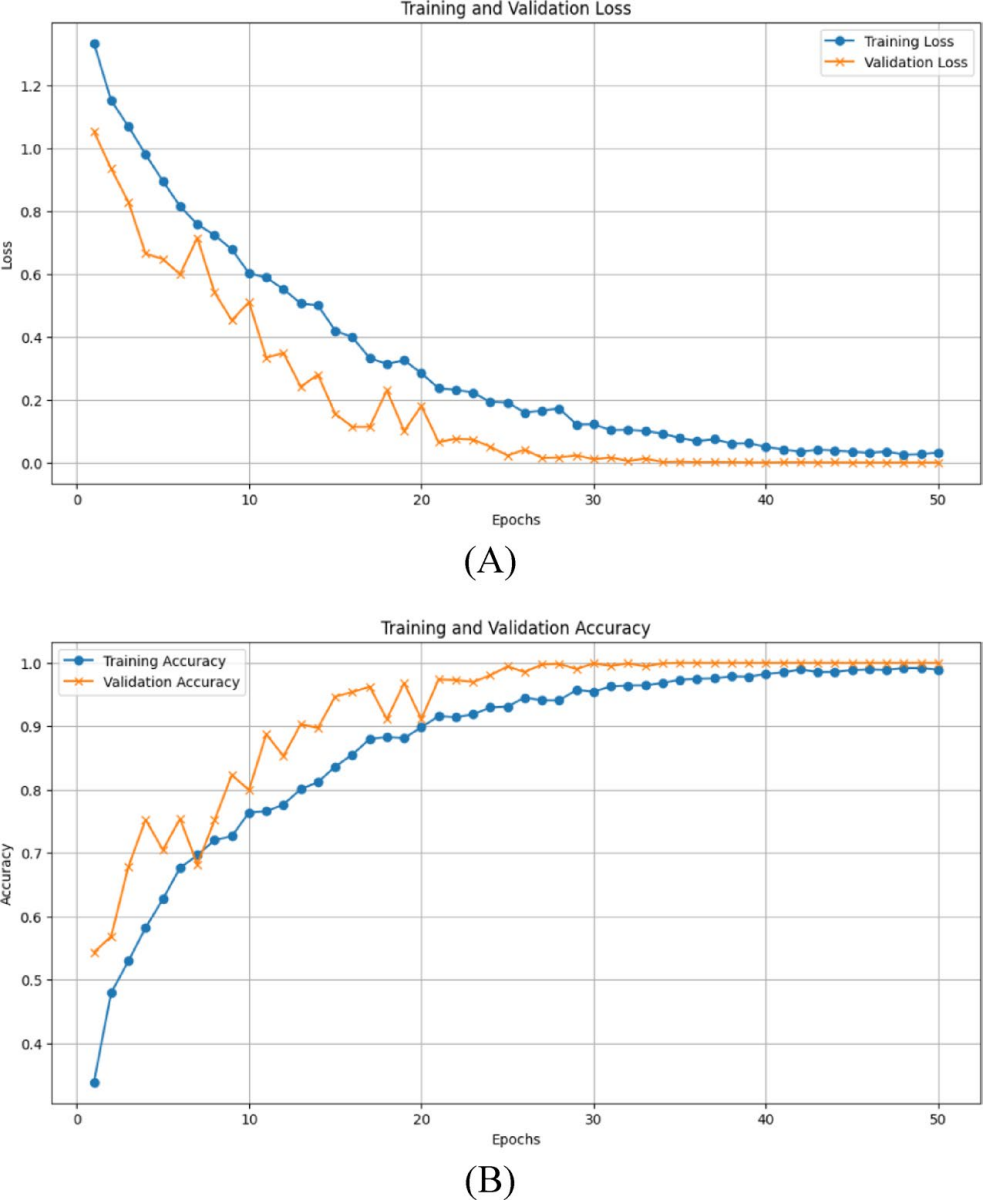
**(A)**The performance curve of training and validation loss, **(B)** The performance curve of training and validation accuracy, of the proposed model.

The optimal loss and accuracy curve produced by our suggested model when the Adam optimizer is used with a 0.001 learning rate is shown in Fig. 4.

**Fig. 5:**
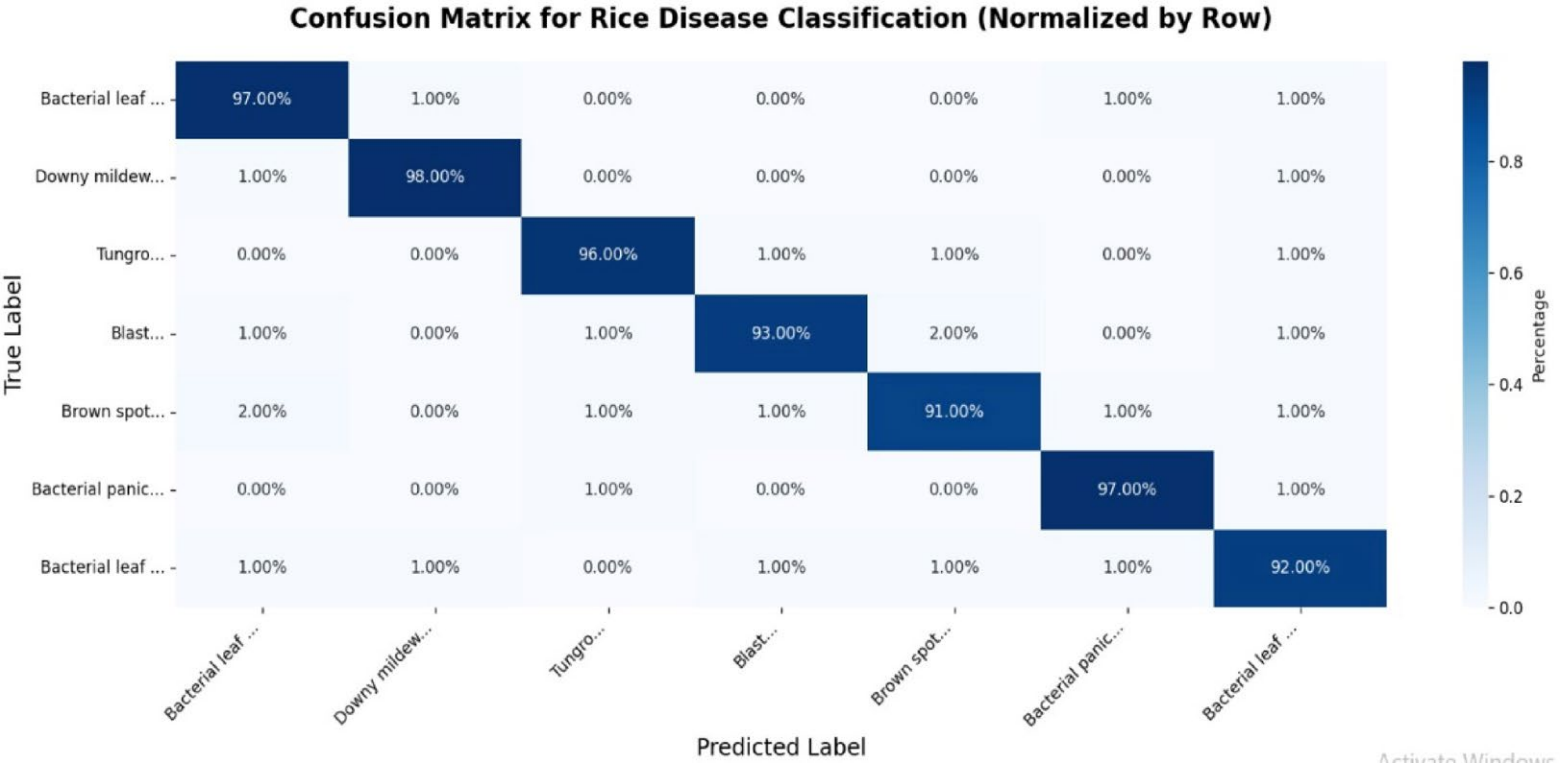
The confusion matrix for the our model.

Figure 5 shows the performing model’s confusion matrix. By examining the important diagonal elements of the confusion matrix, we may tell which photos in that Figure were correctly classified. A greater recall number suggests that the results are dispersed more evenly across classes. As a result, the model performed better with the image dataset.

Our proposed method achieved an average testing accuracy of 97.9%. Analysis of the normalized confusion matrix reveals that, apart from brown spot disease, all other disease categories are detected with high accuracy, demonstrating the effectiveness of our model in distinguishing between different disease classes.

Table 6 presents a comparison between our model and previous related studies. This comparison highlights that most prior research has focused on a narrower range of disease types.

**Table 6:** Comparison between our other model and existent works.

Authors	Proposed Method	Dataset	Performance
Saputra, A.D. et al^36^.	DenseNet121 DenseNet169 DenseNet201	Rice Leaves	94% 89% 92%
Krishnamoorthy et al^43^.	CNN	3 types of disease classes	95.67%
Our proposed model	DenseNet121	12 classes contain 19,211 sample of images	97.9%

### Cross-validation analysis

Cross-Validation analysis performed a thorough 5-fold to assess the stability and robustness of our suggested DenseNet121 model. This thorough evaluation process guarantees that the given performance metrics reflect consistent model behavior across many data divisions rather than being byproducts of a specific train-test split. The dataset was split into five equal folds at random; four of the folds were utilized for training, and each fold was used as the validation set once. To get a comprehensive cross-validation evaluation, this procedure was carried out five times.

The comprehensive 5-fold cross-validation results are shown in Table 7, which shows that the model’s performance is consistent across various data divisions. With accuracy ranging from 97.2% to 98.4% across all folds and a mean accuracy of 97.8% ± 0.42%, the results demonstrate exceptional stability. Our model is not overfitted to any specific subset of the data, as evidenced by the low standard deviation (σ = 0.42%), which shows little variation in performance. The robustness of our technique is further validated by the consistent performance of precision, recall, and F1-score metrics across all folds, with standard deviations below 0.5% for all metrics.

**Table 7:** 5-Fold cross-validation results.

Fold	Accuracy (%)	Precision (%)	Recall (%)	F1-Score (%)
Fold 1	97.6	96.0	97.8	96.9
Fold 2	98.1	96.5	98.2	97.3
Fold 3	97.2	95.8	97.5	96.6
Fold 4	98.4	96.8	98.3	97.5
Fold 5	97.7	96.1	97.9	97.0
Mean ± SD	97.8 ± 0.42	96.2 ± 0.37	97.9 ± 0.32	97.1 ± 0.36

### Statistical significance analysis

Extensive statistical analysis was performed by using paired t-tests and calculated 95% confidence intervals for all important performance indicators in order to thoroughly confirm the statistical significance of our model’s performance gains in comparison to baseline approaches. The statistical analysis offers compelling proof of our DenseNet121 implementation’s advantages over other architecture documented in the literature.

The 95% confidence intervals for each evaluation metric, derived from the five cross-validation folds, are shown in Table 8. Our results’ stability and dependability are further supported by the small confidence intervals. The 95% confidence interval (CI) for accuracy is [97.2%, 98.4%], meaning that can be 95% certain that the genuine population accuracy is within this range. The robustness of our results is also supported by the narrow confidence bounds shown by precision (95% CI: [95.7%, 96.7%]), recall (95% CI: [97.4%, 98.4%]), and F1-score (95% CI: [96.6%, 97.6%]).

**Table 8:** 95% confidence intervals and statistical Significance.

Metric	Mean (%)	95% CI	*p*-value
Accuracy	97.8	[97.2, 98.4]	< 0.001
Precision	96.2	[95.7, 96.7]	< 0.001
Recall	97.9	[97.4, 98.4]	< 0.001
F1-Score	97.1	[96.6, 97.6]	< 0.001

### Discussion

The application of DenseNet121 to plant disease datasets represents a significant advancement in the field of agricultural technology and machine learning. This study aimed to explore the effectiveness of DenseNet121, a convolutional neural network architecture known for its efficiency in feature extraction and representation learning, in the context of plant disease classification. The experiments conducted yielded promising results, but they also raised several important considerations regarding the implementation, performance, and implications of using such models in real-world agricultural scenarios.

One of the primary findings of this study is that DenseNet121 can be effectively adapted beyond previously reported single‑crop disease classification tasks, such as rice disease identification, to address a broader and more practical plant‑stress recognition problem. While DenseNet121 has been successfully applied to rice disease classification in earlier studies, our work differs in task formulation and application scope. Specifically, we evaluate the model’s ability to discriminate between two visually similar plant diseases and one pest‑induced damage category, which introduces a higher level of complexity than disease‑only classification.

The dense connectivity pattern of DenseNet121 enhances feature reuse and gradient propagation, which is particularly advantageous for capturing subtle visual differences between pathogen‑induced symptoms and insect‑related damage under field conditions. This distinction is critical in real agricultural scenarios, where farmers must first determine whether observed symptoms are caused by disease or pest activity before selecting appropriate management strategies. Our results, therefore, extend previous DenseNet121‑based studies by demonstrating its robustness in a multi‑stress classification setting, rather than merely confirming its performance on a single‑crop disease dataset.

Furthermore, this study emphasizes that model performance is not solely attributable to the architecture itself, but also depends on carefully curated, diverse field data and explicit stress category definitions, which are essential for reliable deployment in real‑world agricultural applications.

The datasets used in this study were sourced^42^, including images of different rice disease species and associated diseases. This diversity is vital for training a robust model capable of generalizing well to unseen data. However, the potential for overfitting remains a concern, particularly when the model encounters images that are significantly different from those in the training set. Future research should focus on augmenting datasets and employing techniques such as transfer learning to enhance model robustness. Additionally, the inclusion of more diverse datasets that encompass various environmental conditions, lighting, and backgrounds could further improve the model’s performance in real-world applications.

## Conclusion and future work

The application of DenseNet121 to plant disease datasets has demonstrated significant potential for improving disease classification and management in agriculture. While the results are promising, further research is needed to address the challenges of data diversity, model interpretability, ethical considerations, and interdisciplinary collaboration. By continuing to explore these avenues, we can harness the power of deep learning to create impactful solutions that support sustainable agricultural practices and enhance food security worldwide. Moreover, the integration of DenseNet121 with other technologies, such as mobile applications or drones, could revolutionize the way plant diseases are monitored and managed. The real-time processing capabilities of deep learning models can be harnessed to develop tools that provide immediate feedback to farmers, enabling them to take timely actions to mitigate the impact of diseases. However, this integration will require careful consideration of computational resources and the deployment of models in resource-limited settings. Strategies to optimize the model for mobile platforms or edge devices will be essential to ensure accessibility for farmers in various regions.

Finally, the findings of this study underscore the need for interdisciplinary collaboration in addressing the challenges of plant disease management. The intersection of machine learning, agronomy, and plant pathology is a fertile ground for innovation, and fostering partnerships among these fields can lead to the development of more effective and practical solutions. Engaging with agricultural stakeholders, including farmers, agricultural extension services, and policymakers, will be crucial in translating these technological advancements into actionable strategies that enhance food security and sustainability.

Limitations and Future Directions: While our results are promising, several limitations warrant consideration. The model performance may be affected by image quality variations in field conditions, including poor lighting, motion blur, and occlusion by environmental factors. The dataset, though comprehensive, represents controlled capture conditions and may not fully encompass the geographic and environmental diversity of global rice cultivation. Certain disease pairs with overlapping visual symptoms (e.g., brown spot and blast) show minor confusion, suggesting the need for multi-modal diagnostic approaches incorporating temporal progression patterns or spectral imaging. Future work should address these limitations through validation on diverse field-collected datasets, development of uncertainty quantification mechanisms, and integration with farmer feedback systems to enhance model robustness and practical utility.

## Data Availability

The data presented in this study are available in Kaggle^42^.
